# Effect of Infliximab on the UVB-Induced Apoptosis of Keratinocytes Infected by HPV38 E6/E7

**DOI:** 10.1155/2013/907189

**Published:** 2013-02-25

**Authors:** François Aubin, Tarik Gheit, Jean Luc Prétet, Massimo Tommasino, Christiane Mougin

**Affiliations:** ^1^Molecular and Cell Biology Laboratory, Université de Franche Comté, EA3181, SFR FED4234, Besançon, France; ^2^Department of Dermatology, Centre Hospitalier Universitaire, 25030 Besançon, France; ^3^Department of Infections and Cancer Biology, IARC, Lyon, France

## Abstract

The question of the effect of anti-TNF-alpha in skin carcinogenesis is especially relevant in view of the increased use of these drugs for the treatment of autoinflammatory immune diseases. Since ultraviolet radiation and human papillomavirus are involved in skin carcinogenesis, we wished to investigate the effect of TNF-alpha antagonists on the UVB-induced apoptosis of keratinocytes infected by HPV38. Our results indicate that anti-TNF agent, infliximab, does not contribute to the survival of HPV38-transduced keratinocytes with UVB-induced DNA damages.

## 1. Introduction

Tumor necrosis factor-alpha (TNF-*α*) is one of the main mediators of skin and mucosa inflammation and has a potent antiproliferative effect on normal epithelial cells. Numerous studies indicate that TNF-*α* may influence the fate of HPV-infected cells and suggest that HPV-mediated TNF resistance is a key event in the multistep process leading to cervical cancer [1].

Although several epidemiological studies have reported an increased risk of nonmelanoma skin cancer in patients treated with anti-TNF agents, the relationship remains uncertain [2]. The question of the effect of anti-TNF-*α* in skin carcinogenesis is, thus, especially relevant in view of the increased use of these drugs for the treatment of autoinflammatory immune diseases.

Besides ultraviolet (UV) irradiation which is the most important risk factor involved in the development of NMSC, cutaneous beta-HPV infection is also considered as an important cofactor [3]. Similar to the high-risk alpha mucosal HPV types, E6 and E7 oncoproteins from certain beta-HPV types target p53- and pRb-regulated pathways and display transforming activities. In particular, E6 and E7 from the beta HPV38 are able to immortalize keratinocytes that are natural hosts of the virus [4, 5]. To our knowledge, there are no available data on the interactions between cutaneous HPV infection and anti-TNF agents. 

Since anti-TNF agents are also associated with an increased risk of viral infections [6], we wished to investigate the effect of an anti-TNF-*α* agent, infliximab, on the UVB-induced apoptosis of keratinocytes infected by HPV38.

## 2. Materials and Methods

HaCaT keratinocytes and HaCaT keratinocytes transduced with empty vector pLXSN and with pLXSN-HPV38 E6/E7 vector [7] were UVB irradiated (10 mJ/cm^2^) to induce apoptosis. To assess the role of TNF-*α*, 100 ng/mL of TNF-*α* (Sigma Aldrich, Saint Quentin Fallavier, France) and 20 *μ*g/mL of infliximab (MSD, France) were added after UVB irradiation. Apoptosis was evaluated by flow cytometry (annexin V/propidium iodide labelling), fluorescence microscopy and PARP, and cleaved caspase-3 western blot. Soluble TNF-alpha was measured by ELISA (Human TNF-*α* ELISA MAX Deluxe, BioLegend, Ozyme, Saint Quentin, France) in the supernatants. 

## 3. Results

**Figure 1:**
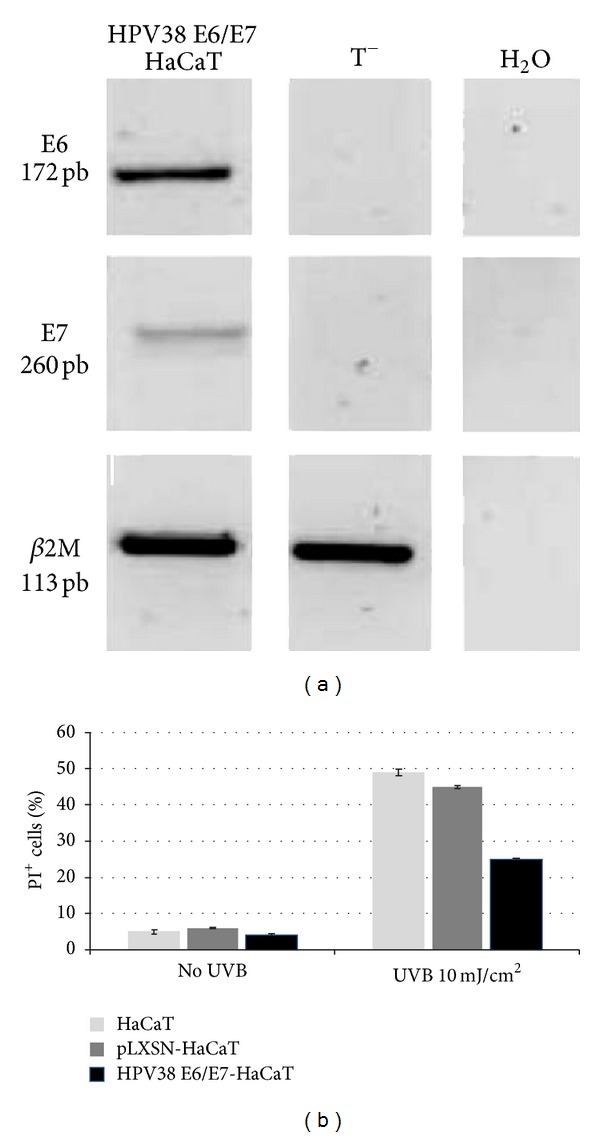
Influence of HPV38 E6/E7 expression on UVB-induced apoptosis. (a) Transduction efficiency was confirmed by RT-PCR analysis on total RNA extracted from HaCaT cells transduced with PLXSN-HPV38 E6/E7 vector. HaCaT cells transduced with the empty vector pLXSN (T−) were used as negative controls, respectively. *β*2M:*β*2 microglobulin gene. (b) HaCaT cells transduced by empty vector pLXSN or with pLXSN-HPV38 E6/E7 vector were irradiated with UVB (10 mJ/cm²) and harvested 24 hours after irradiation. Then, cells were resuspended in propidium iodide (PI+ cells) for analysis by flow cytometry to evaluate the percentage of cells with fragmented DNA.

The gene expression of E6 and E7 in HaCaT cell lines was confirmed by RT-PCR analysis (Figure 1(a)). To ensure that sub-G1 population was representative of apoptotic cells, we used Hoechst staining and TUNEL assays which showed an increasing cell population with nuclear condensation and DNA fragmentation in a time-dependent manner (data not shown). Furthermore, immunoblot analysis detected cleavage of PARP and cleaved caspase-3 following UVB irradiation in both empty vector pLXSN and HPV38 E6/E7-HaCaT cells (data not shown).

**Figure 2:**
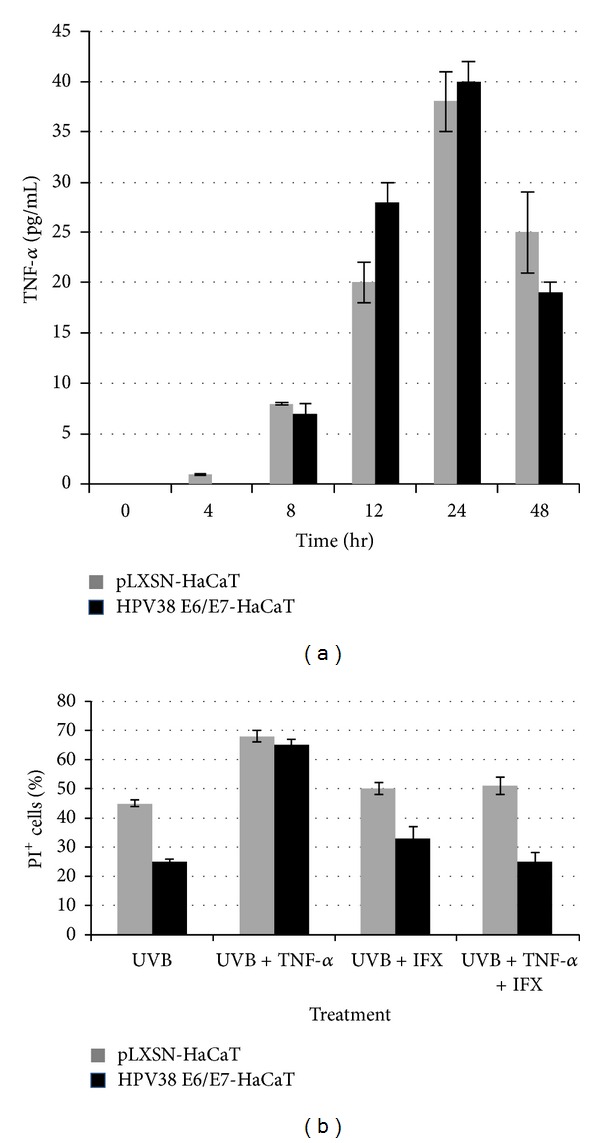
Role of TNF-*α* in UVB-induced apoptosis of HPV38 E6/E7 HaCaT cells. (a) HaCaT cells transduced by empty vector PLXSN or with pLXSN-HPV38 E6/E7 vector were irradiated with UVB (10 mJ/cm²) and harvested 24 hours after irradiation. Soluble TNF-*α* was measured by ELISA test (Human TNF-*α* ELISA MAX Deluxe, BioLegend, Ozyme, Saint Quentin, France) in the supernatant. (b) pLXSN-HaCat and HPV38 E6/E7-HaCaT cells were irradiated in phosphate-buffered saline with UVB (10 mJ/cm^2^) and then incubated in Dulbecco's Modified Eagles medium with TNF-*α* (100 ng/mL) and/or infliximab (20 ng/mL) for 24 h. Subsequently, the cells were fixed and stained with propidium iodide (PI), and the percentage of sub-G1 cell population was detected by flow cytometry. The experiments were performed at least three times independently in duplicate. The error bars indicate standard deviation.

As expected [7], exposure to UVB irradiation induced HaCaT-pLXSN and HPV38 E6/E7-HaCaT cells apoptosis. However, the percentage of apoptotic HPV38 E6/E7 cells induced by UVB was 2-fold lower than those observed in HaCaT-pLXSN cells (Figure 1(b)). UVB irradiation was associated with a similar release of TNF-*α* in the supernatants from both pLXSN and HPV38 E6/E7-HaCaT cells (Figure 2(a)). Addition of infliximab (100 ng/mL) did not modulate UVB-induced apoptosis of both pLXSN-HaCaT and HPV38 E6/E7-HaCaT cells. Exposure to exogenous TNF-*α* resulted in a significant increase of UVB-induced apoptosis with no significant difference in both pLXSN-HaCaT and HPV38 E6/E7-HaCaT cells. Furthermore, inhibition of TNF by the addition of infliximab (20 *μ*g/mL) restored UVB-induced apoptosis level (Figure 2(b)).

## 4. Discussion

Previous studies have already demonstrated that UVB light induces the release of TNF-*α* by keratinocytes [8] and that TNF-*α* increases apoptosis in both normal human keratinocytes and premalignant HaCaT cells [9, 10]. Furthermore, polyclonal rabbit anti-TNF-*α* antibody has been shown to partially reduce UVB-induced apoptosis in HaCaT cells [10]. Our results confirm recent data [11] demonstrating that HPV38 E6 and E7 expression in human primary keratinocytes was associated with a low level of UVB-induced apoptosis through the expression of NF-*κ*B- regulated survival factor genes favoring the survival of keratinocytes. Furthermore, the fact that we observed a lower apoptotic rate in HPV38 E6/E7 cells than in HaCaT-vector cells despite similar release of TNF-*α* after UVB exposure further suggests that TNF-*α* is only partially involved in UVB-induced apoptosis as previously suggested [10]. However, it should be noticed that the addition of high dose of TNF-*α* (100 ng/mL) restored a similar level of apoptosis in both pLXSN-HaCaT and HPV38 E6/E7-HaCaT cells. This suggests that exogenous TNF-*α* is able to suppress the inhibitory effect of HPV38 E6 and E7 expression on UVB-induced apoptosis of keratinocytes. Moreover, our results demonstrate that anti-TNF-*α* agent, infliximab, does not modulate UVB-induced apoptosis of both HaCaT cells and HPV38 E6/E7-infected keratinocytes. In a previous *ex vivo* study, Gambichler et al. [12] observed that etanercept, a recombinant human TNF-*α* receptor fusion protein, had no effect on the number of caspase-3 apoptotic cells present in skin biopsies of psoriasis patients treated with UVB and etanercept. In contrast, Faurschou et al. [13] observed an unexpected increase of UVB-induced apoptosis in HaCaT keratinocytes exposed to infliximab despite enhanced G2/M cell cycle checkpoint and TNF-*α*-induced apoptosis. The authors suggested a reverse signalling pathway via membrane-bound TNF-*α*.

It may be argued that in our study infliximab dose (20 *μ*g/mL) was too low to overcome the endogenous TNF-*α* production. However, the dose tested was still able to neutralize exogenous TNF-*α* (100 ng/mL) which induced a 2-fold apoptotic rate compared with UVB alone (Figure 2). It should also be noticed that endogenous production of TNF-*α* by UVB-irradiated keratinocytes was not altered by HPV38 E6/E7 infection. 

Altogether, our results indicate that anti-TNF agent, infliximab, does not contribute to the survival or the elimination of HPV38-transduced keratinocytes with UVB-induced DNA damages. Other TNF-independent pathways may be involved [10, 11]. These results are quite encouraging in view of the increased use of anti-TNF-*α* agents in different autoinflammatory immune diseases.
